# Epidemiological characteristics and risk factors for cystic and alveolar echinococcosis in China: an analysis of a national population-based field survey

**DOI:** 10.1186/s13071-023-05788-z

**Published:** 2023-06-03

**Authors:** Tian Ma, Qian Wang, Mengmeng Hao, Chuizhao Xue, Xu Wang, Shuai Han, Qian Wang, Jiangshan Zhao, Xiao Ma, Xianglin Wu, Xiaofeng Jiang, Lei Cao, Yaming Yang, Yu Feng, Quzhen Gongsang, Jürgen Scheffran, Liqun Fang, Richard James Maude, Canjun Zheng, Fangyu Ding, Weiping Wu, Dong Jiang

**Affiliations:** 1 Institute of Geographic Sciences and Natural Resources Research, Chinese Academy of Sciences, Beijing, 100101 China; 2 College of Resources and Environment, University of Chinese Academy of Sciences, Beijing, 100049 China; 3 Mahidol Oxford Tropical Medicine Research Unit, Faculty of Tropical Medicine, Mahidol University, Bangkok, Thailand; 4 Centre for Tropical Medicine and Global Health, Nuffield Department of Medicine, University of Oxford, Oxford, UK; 5 National Institute of Parasitic Diseases, Chinese Center for Disease Control and Prevention, Shanghai, 200025 China; 6 Sichuan Provincial Center for Disease Control and Prevention, Chengdu, Sichuan China; 7 Xingjiang Uyghur Autonomous Region Center for Disease Control and Prevention, Urumqi, Xinjiang China; 8 Qinghai Institute for Endemic Disease Prevention and Control, Xining, Qinghai China; 9 Ningxia Center for Disease Control and Prevention, Yinchuan, Ningxia China; 10 Inner Mongolia Autonomous Region Center for Diseases Control and Prevention, Hohhot, Inner Mongolia China; 11 Shaanxi Provincial Center for Disease Control and Prevention, Xi’an, Shaanxi China; 12 Yunnan Institute of Parasitic Diseases, Puer, Yunnan China; 13 Gansu Provincial Center for Disease Control and Prevention, Lanzhou, Gansu China; 14 Tibet Center for Diseases Control and Prevention, Lhasa, Tibet China; 15 Institute of Geography, Center for Earth System Research and Sustainability, University of Hamburg, 20144 Hamburg, Germany; 16 State Key Laboratory of Pathogen and Biosecurity, Beijing Institute of Microbiology and Epidemiology, Beijing, China; 17 Harvard TH Chan School of Public Health, Harvard University, Boston, USA; 18 The Open University, Milton Keynes, UK; 19 Chinese Center for Disease Control and Prevention, Beijing, China; 20 Key Laboratory of Carrying Capacity Assessment for Resource and Environment, Ministry of Natural Resources, Beijing, China

**Keywords:** Cystic echinococcosis, Alveolar echinococcosis, Epidemiological characteristics, Risk factors, China

## Abstract

**Background:**

Human cystic and alveolar echinococcosis are neglected tropical diseases that WHO has prioritized for control in recent years. Both diseases impose substantial burdens on public health and the socio-economy in China. In this study, which is based on the national echinococcosis survey from 2012 to 2016, we aim to describe the spatial prevalence and demographic characteristics of cystic and alveolar echinococcosis infections in humans and assess the impact of environmental, biological and social factors on both types of the disease.

**Methods:**

We computed the sex-, age group-, occupation- and education level-specific prevalences of cystic and alveolar echinococcosis at national and sub-national levels. We mapped the geographical distribution of echinococcosis prevalence at the province, city and county levels. Finally, by analyzing the county-level echinococcosis cases combined with a range of associated environmental, biological and social factors, we identified and quantified the potential risk factors for echinococcosis using a generalized linear model.

**Results:**

A total of 1,150,723 residents were selected and included in the national echinococcosis survey between 2012 and 2016, of whom 4161 and 1055 tested positive for cystic and alveolar echinococcosis, respectively. Female gender, older age, occupation at herdsman, occupation as religious worker and illiteracy were identified as risk factors for both types of echinococcosis. The prevalence of echinococcosis was found to vary geographically, with areas of high endemicity observed in the Tibetan Plateau region. Cystic echinococcosis prevalence was positively correlated with cattle density, cattle prevalence, dog density, dog prevalence, number of livestock slaughtered, elevation and grass area, and negatively associated with temperature and gross domestic product (GDP). Alveolar echinococcosis prevalence was positively correlated with precipitation, level of awareness, elevation, rodent density and rodent prevalence, and negatively correlated with forest area, temperature and GDP. Our results also implied that drinking water sources are significantly associated with both diseases.

**Conclusions:**

The results of this study provide a comprehensive understanding of geographical patterns, demographic characteristics and risk factors of cystic and alveolar echinococcosis in China. This important information will contribute towards developing targeted prevention measures and controlling diseases from the public health perspective.

**Graphical Abstract:**

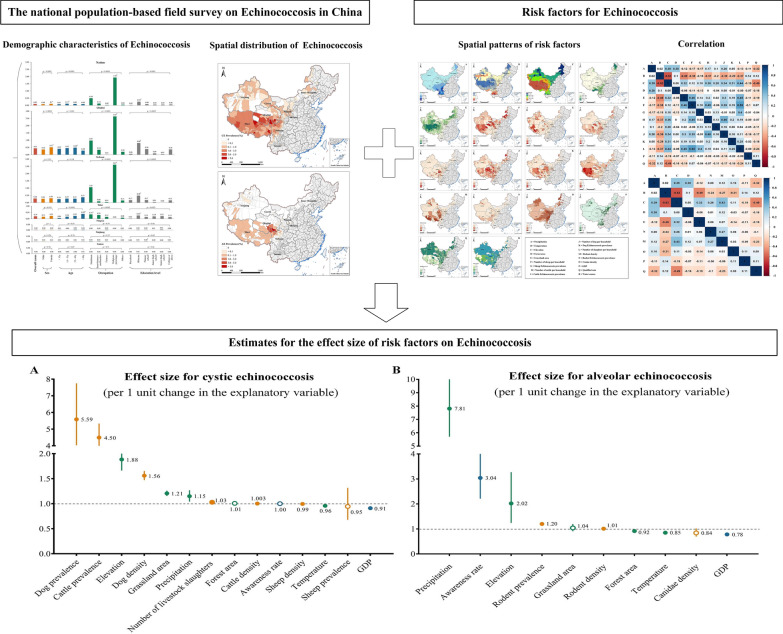

**Supplementary Information:**

The online version contains supplementary material available at 10.1186/s13071-023-05788-z.

## Background

Echinococcosis is a neglected zoonotic disease caused by larval stages of cestodes belonging to the genus *Echinococcus* [1, 2]. Cystic echinococcosis (CE) and alveolar echinococcosis (AE), the two most common forms of echinococcosis in humans, are caused by the tapeworms *Echinococcus granulosus* and *Echinococcus multilocularis*, respectively [1]. Specifically, CE is mainly transmitted through domestic cycles (herbivores—dogs), while AE is primarily transmitted through sylvatic ones (small mammals—wild carnivores) [1]. Humans usually get infected with both diseases accidentally as intermediate hosts when they ingest eggs of *Echinococcus* spp. transmitted from infected definitive hosts or come into contact with a contaminated environmental source [3]. Both CE and AE are deemed severe life-threatening diseases, of which CE is considered a disabling disease, while AE is a lethal disease with a 10-year fatality rate of 94% for untreated or inadequately managed patients [3, 4].

Echinococcosis has been listed as one of the 20 neglected tropical diseases recognized by the WHO and prioritized by the WHO for control efforts [5]. CE is found in all continents except Antarctica, with high endemicity in western China, Central Asia, South America, the Mediterranean countries and eastern Africa [2]. It is estimated that there are at least 188,000 new CE cases worldwide per year, causing a loss of at least 1 million disability-adjusted life-years (DALYs), of which China accounts for 40% of the total [6]. By contrast, AE is considered to be confined to the northern hemisphere, primarily distributed in western China, central Asia, Russia, the Baltic region and North America [2], with an estimated > 18,000 new cases annually, resulting in a loss of 670,000 DALYs [7], among which 91% of cases and 95% of DALYs are estimated to have occurred in China [7].

As one of the most endemic regions for both CE and AE, China has suffered from these diseases for a long time. In 1905, the first human infected with CE was documented in Qingdao, Shandong Province [8]. From the 1950s to the early 2000s, at least 35,000 cases were treated surgically in China [9], and subsequent observations indicated that human CE showed an obvious increasing trend [10]. The first human case of AE was recorded in 1965 in Urumqi, Xinjiang Province [11]. At first, the disease was considered to be sporadic in China but, unexpectedly, convincing reports of human AE cases then appeared successively in most western provinces [12, 13]. In response, a national control program for echinococcosis was launched in China in 2005, including activities of health education, sanitation improvement, ultrasound screening of the human population, surgical interventions and dog deworming. Consequently, a remarkable decrease in human echinococcosis was observed, falling from 1.08% prevalence in 2004 to 0.24% in 2012 [14]. However, the results of a national epidemiological survey carried out between 2012 and 2016 suggested that echinococcosis still remains a grave threat in western China, with statistics showing that about 170,000 people have been infected with echinococcus and an estimated 50 million people were at risk of contracting the disease [15].

Given the severe situation in China, some previous studies have examined the prevalence distribution and risk factors for CE or AE in selected provinces [16, 17]. However, to our knowledge, there has been no comprehensive and comparable descriptions of prevalence characteristics for both CE and AE across China. In addition, no research has been conducted to systematically and quantitatively analyze the environmental, biological and socio-economic risk factors for both diseases in China. To fill these knowledge gaps, in this study our aim is to describe demographic characteristics of people with echinococcosis in China, to map the prevalence of both diseases at high spatial resolution and to assess the impact of environmental, biological and social factors on both main forms of the disease. To do this, we will analyze data from the largest national epidemiological survey on echinococcosis between 2012 and 2016. The information from our study can help to uncover populations and areas at high risk of contracting echinococcosis and elucidate potential risk factors associated with it, which can be beneficial for targeted prevention and control of the disease.

## Methods

### National echinococcosis survey

The national echinococcosis survey designed by the Chinese Center for Disease Control and Prevention (China CDC) was conducted between 2012 and 2016 [15]. Counties with suspected local echinococcosis cases and suitable conditions for disease transmission in nine provinces and autonomous regions (Inner Mongolia, Sichuan, Tibet, Gansu, Qinghai, Ningxia, Yunnan, Shaanxi, and Xinjiang) in China were included in the survey. Sixteen villages in each included county were randomly selected, from each of which 200 residents aged > 1 year were examined by B-ultrasonography, which was accompanied by a serological test for those found to have suspected echinococcosis. Cases were diagnosed and classified according to the “Diagnostic criteria for echinococcosis” of China (Standard WS 257–2006; China Disease Prevention and Control Center of Parasitic Diseases Control and Prevention, PRC Ministry of Health, Beijing, China), which is in line with the WHO classification. Demographic information on participants’ gender, age, occupation and education level was also collected. In addition, echinococcosis prevalence in local animal hosts was investigated. Twenty households with dogs were randomly selected from each village, and one fresh dog fecal sample was collected from each household and subsequently tested for echinococcus antigen by enzyme-linked immunosorbent assay (ELISA). During the slaughtering season, about 1000 sheep or 500 cattle were selected in each county for examination for *Echinococcus* infection by visual inspection and external palpation of internal organs (i.e. livers, lung, and other organs). In AE endemic counties, > 1000 adult rodents were captured around residences with AE patients or regions with dogs. A visceral biopsy was performed on the rodents to examine lesions caused by *E. multilocularis* and identified by microscopy. For each registered household, a questionnaire (Additional file 1: Questionnaire 1) was designed to obtain information on the number of livestock (cattle, sheep, dogs) raised, the number of livestock slaughtered and the drinking water sources per household. A second questionnaire ((Additional file 1: Questionnaire 2) was used to assess the awareness of prevention and control measures against echinococcosis of local residents, and their level of awareness was rated on a scale of 0–100 (a score of 60 indicated adequate awareness).

### Spatial covariates and data preprocessing

We assembled a range of environmental, biological and socio-economic factors thought to affect the prevalence of echinococcosis as potential explanatory covariates in this study (Table 1). We also generated the average temperature and precipitation data at a spatial resolution of 1 km by ANUSPLIN-SPLINA software based on the daily climate records of weather stations from the China Meteorological Data Service Center (CMDS) (http://data.cma.cn) and then calculated the average value of precipitation and temperature within each county. Landcover data with a spatial resolution of 30 m was downloaded from the website of GlobeLand30 (http://globeland30.org), then the total area of grassland and forest was summed at the county level. The gridded elevation and gross domestic product (GDP) data (resolution: 1 × 1 km) were obtained from the Data Center for Resources and Environmental Sciences, Chinese Academy of Science (RESDC) (http://www.resdc.cn). Canidae density data were extracted from gridded wild animal density data obtained from the Socioeconomic Data and Applications Center (SEDAC) (https://sedac.ciesin.columbia.edu). We then calculated county-level elevation, GDP and Canidae density data by averaging the gridded value in each county. Moreover, based on data in the national echinococcosis survey, we calculated the number of sheep, cattle and dogs raised per household and the number of rodents captured within each county as county-level animal density data. We also calculated county-level echinococcosis prevalence in animal hosts (dogs, cattle, sheep and rodents) as: number of positive animals within each county/number of examined animals within each county. The average number of livestock slaughtered per household was calculated at the county level. The awareness rate of each county was calculated: number of people who passed the test/number of people who took the test. The proportion of each drinking water source in each county was calculated, and the most common water source was considered to be the drinking water source of this county. The spatial distributions of these covariates are shown in Additional file 2: Figure S1.

**Table 1:** Spatial covariates included in this analysis

Factors	Variables	Data sources
Environmental	Precipitation	China Meteorological Data Service Center (CMDS)
	Temperature	
	Grass area	GLOBELAND30
	Forest area	
	Elevation	Consultative Group on International Agricultural Research (CGIAR)
Biological	Sheep density	The Chinese Center for Disease Control and Prevention (China CDC)
	Cattle density	
	Dog density	
	Sheep prevalence	
	Cattle prevalence	
	Dog prevalence	
	Number of livestock slaughters	
	Rodent density	
	Rodent prevalence	
	Canidae density	Socioeconomic Data and Applications Center (SEDAC)
Socio-economic	Gross domestic product	The Data Center for Resources and Environmental Sciences, Chinese Academy of Science (RESDC)
	Awareness rate	The Chinese Center for Disease Control and Prevention (China CDC)
	Water source	

### Statistical analysis

The prevalence of CE and AE was calculated and mapped at the province, city and county levels. We also computed sex-specific, age group-specific, occupation-specific and education level-specific prevalence of both diseases at national and sub-national levels. All prevalence estimates were calculated by the number of diagnosed cases divided by the number examined. Significant differences in sex, age, occupation and education level for echinococcosis were assessed by the Chi-square test or Fisher’s exact test. We also estimated the odds ratio (OR) to describe the strength of the association of demographic variables (i.e. sex, age, occupation and education level) with echinococcosis (Additional file 3: Text S1). To investigate drivers of echinococcosis heterogeneity in China, we formulated a multivariable generalized linear model based on a combination of county-level echinococcosis cases and a range of environmental, biological and socio-economic factors (Additional file 4: Text S2). We constructed separate models for CE and AE considering their differences in transmission cycles and animal hosts. Spearman correlation tests were conducted to evaluate the correlations between covariates, and collinearity between variables was tested by a variance inflation factor (VIF) (Additional file 2: Figures S2 and S3). All statistical tests were two-sided, and *P* < 0.05 was considered to indicate statistical significance. Prevalence estimation, descriptive statistics, OR computation and model fitting were carried out using R, version 3.3 (Foundation for Statistical Computing, Vienna, Austria). Spatial analysis and mapping were done using ArcGIS 10.6 (Environmental Systems Research Institute Inc. [ESRI], Redlands, CA, USA).

## Results

A total of 1,150,723 residents were selected and included in the national echinococcosis survey between 2012 and 2016, of whom 4161 and 1055 were diagnosed as having CE and AE, respectively. To assess the differences between different types of echinococcosis, we further depicted the demographic characteristics of both CE and AE, building on our previous work [15], which was listed in Table 2. Nationally, sex-specific significant differences were observed for both diseases (*P* < 0.0001), showing that females are on average 1.35-fold (95% confidence interval [CI] 1.27–1.44) and 1.32-fold (95% CI 1.17–1.50) as likely to be infected with CE and AE as males. Among age-specific groups, CE showed increasing prevalence with increasing age (*P* < 0.0001), with a prevalence of 0.19% (age < 15 years), 0.33% (age 15–35 years), 0.35% (age 35–60 years) and 0.52% (age > 60 years), respectively. The oldest group (> 60 years) was almost threefold more likely to be infected with CE than children (< 15 years) (OR 2.82, 95% CI 2.42–3.29). In comparison, for AE, people aged < 15 years had the lowest prevalence (0.07%), with prevalence increasing to its peak in the 15- to 35-year age group (0.11%) and then decreasing in higher age groups. The risk of AE infection in younger (15–35 years) and middle-aged (35–60 years) persons was 1.61- and 1.36-fold higher than in children (< 15 years). Data on the sex- and age-specific prevalences of both echinococcosis are available in Additional file 2: Figures S4, S5. In the assessment of the effect of occupation, religious workers were found to have the highest prevalence for both diseases, with values of 2.56% for CE and 1.93% for AE, followed by herdsmen (CE 1.36%, AE 0.49%). In comparison with other occupations, being a religious worker increased the risk of being infected with both diseases (CE: OR 15.86, 95% CI 11.61–21.66; AE: OR 54.18, 95% CI 36.37–80.72). Similarly, significant associations were observed for herdsmen and CE (OR 8.33, 95% CI 7.49–9.27), and for herdmen and AE (OR 13.42, 95% CI 10.76–16.73). Within education-level groups, both types of echinococcosis were more frequently identified in illiterate people than in literate people (*P* < 0.0001); persons in other education level groups had a 70–95% decreased risk of being infected with echinococcosis compared to persons in the illiterate group.

**Table 2:** Demographic characteristics of cystic echinococcosis and alveolar echinococcosis in China, 2012–2016

Demographic characteristics	No. of total persons examined	Cystic echinococcosis			Alveolar echinococcosis		
		No. of positive persons	Prevalence (%)	Odds ratio (95% CI)	No. of positive persons	Prevalence (%)	Odds ratio (95% CI)
*Sex*							
Male	547,291	1670	0.30	1	429	0.08	1
Female	603,389	2491	0.41	1.35 (1.27–1.44)*	626	0.10	1.32 (1.17–1.50)*
*Age (years)*							
< 15	101,076	188	0.19	1	68	0.07	1
15–35	303,907	1010	0.33	1.79 (1.53–2.09)*	328	0.11	1.61 (1.24–2.08)*
35–60	539,851	1886	0.35	1.88 (1.62–2.18)*	495	0.09	1.36 (1.06–1.76)*
> 60	205,879	1077	0.52	2.82 (2.42–3.29)*	164	0.08	1.18 (0.89–1.57)
*Occupation*							
Herdsman	158,074	2149	1.36	8.33 (7.49–9.27)*	767	0.49	13.42 (10.76–16.73)*
Semi-farmers and -herders	418,73	271	0.65	3.94 (3.37–4.60)*	51	0.12	3.36 (2.38–4.74)*
Farmers	706,713	1295	0.18	1.11 (0.99–1.24)	115	0.02	0.45 (0.34–0.59)*
Religious worker	1,761	45	2.56	15.86 (11.61–21.66)*	34	1.93	54.18 (36.37–80.72)*
Others	242,290	400	0.16	1	88	0.04	1
*Degree of education*							
Preschool	20,277	22	0.11	0.12 (0.07–0.17)*	4	0.02	0.08 (0.03–0.21)*
Illiterate	250,505	2341	0.94	1	639	0.25	1
Primary school	455,134	1254	0.28	0.29 (0.27–0.31)*	344	0.08	0.29 (0.26–0.34)*
Junior high school	315,040	378	0.12	0.13 (0.11–0.14)*	41	0.01	0.05 (0.04–0.07)*
Senior high school	71,882	91	0.13	0.13 (0.11–0.16)*	6	0.01	0.03 (0.01–0.07)*
College or above	37,775	75	0.20	0.21 (0.17–0.26)*	21	0.06	0.22 (0.14–0.33)*
Total	1,150,715	4161	0.36		1055	0.09	

The total number of persons in each category (sex, age, occupation, education) did not equal the total number of persons examined due to missing data

*CI* confidence interval

*Prevalence of echinococcosis was significantly different at *P* < 0.05 between the different groups and the reference group (1) based on the 95% CI. Male, age < 15, other occupation, and illiterate were the reference group in sex, age, occupation, and degree of education groups, respectively

**Fig. 1:**
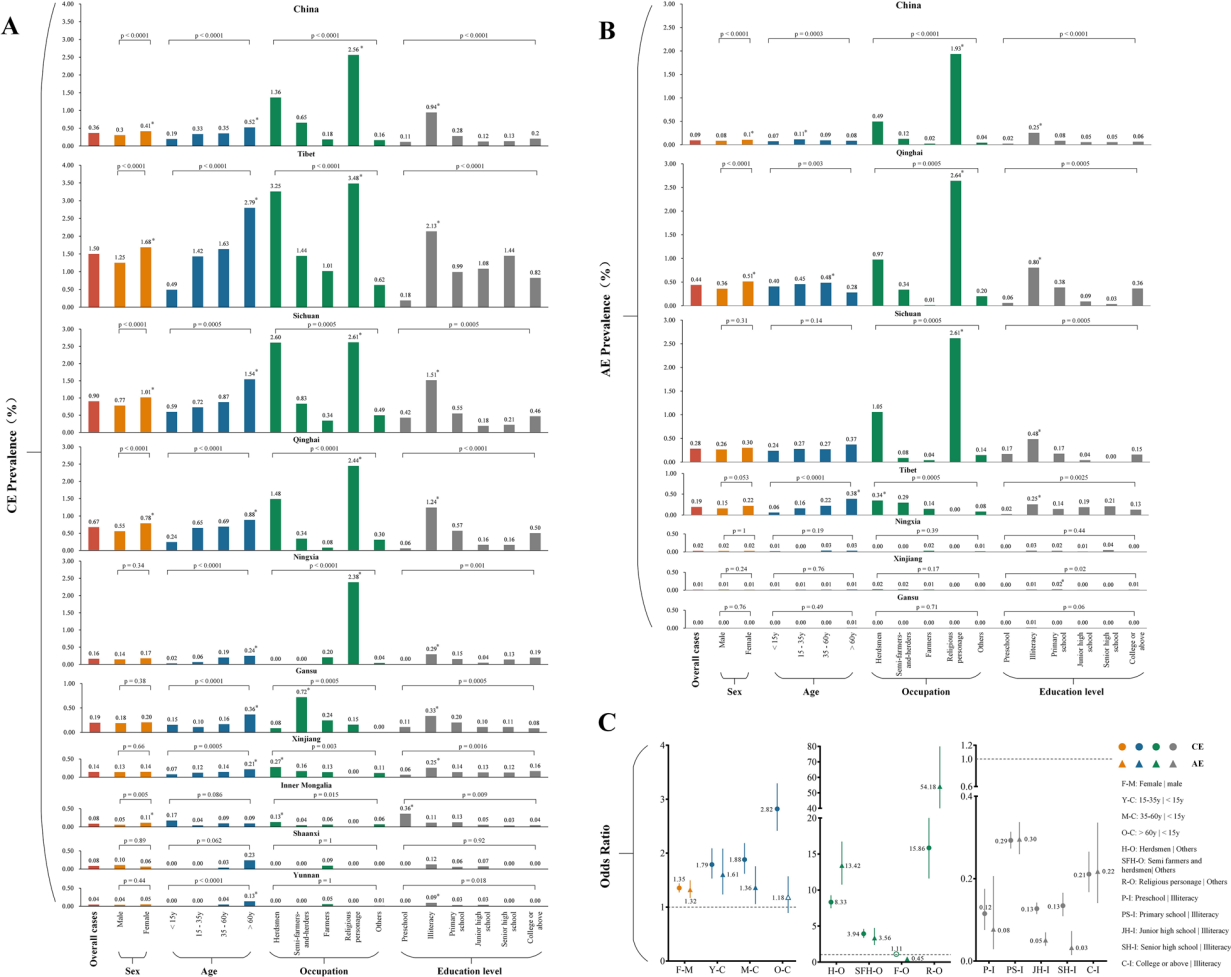
Demographic characteristics of CE and AE in China, 2012–2016. **a** CE, **b** AE, **c** Odds ratio. The lengths of the bars indicate the prevalence in each region. Overall prevalence is given in red; yellow bars indicate prevalence by sex; blue bars, by age group; green bars, by occupation; gray bars by education level. The same bar color indicates the same group. Asterisk indicates a statistically significant difference between the groups at *P* < 0.05 (Chi-square test or Fisher’s exact test were two-sided. AE, Alveolar echinococcosis; CE, cystic echinococcosis

Demographic characteristics of CE and AE varied by province (Fig. 1). The detailed demographic characteristics of echinococcosis for each province are shown in Additional file 5: Tables S1–S9. For CE, the demographic distributions in the provinces of Tibet, Sichuan and Qinghai were consistent with that of China as a whole, as described above. However, in contrast, in Xinjiang and Inner Mongolia, we found a significantly higher prevalence in herdsmen rather than religious workers personage, while in Gansu, we noted that CE was more frequently detected in semi-farmers- and -herdsmen. We detected no statistical association between sex and CE prevalence in the provinces of Ningxia, Gansu, Xinjiang, Shaanxi and Yunnan. For AE, we only observed a statistically significant difference in disease prevalence between genders in Qinghai (*P* < 0.0001), with a higher prevalence in females; in the other provinces, the prevalence of AE was not significantly different between genders. The age-specific pattern for AE differed among provinces. For example, the prevalence of AE in the elderly (> 60 years) was high in Sichuan and Tibet, but in Qinghai province, AE was more frequently identified in those aged 35–60 years. We detected a higher AE prevalence in herdsmen in Tibet, which was different the results from Qinghai and Sichuan, where prevalence was higher in religious workers.

**Fig. 2:**
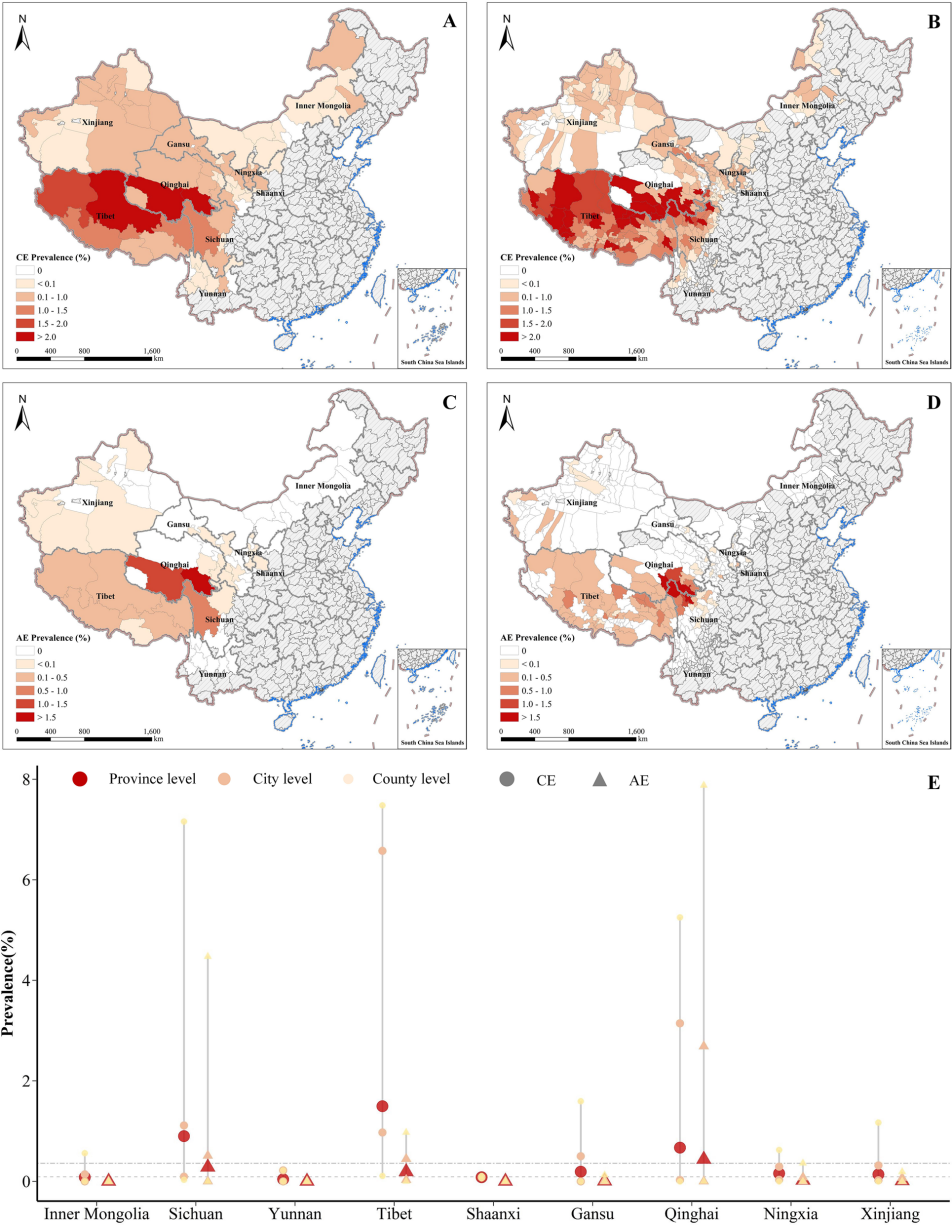
The spatial distribution of prevalence of CE and AE in China.** a**, **b** CE prevalence at the city level (**a**) and county level (**b**). **c**, **d** AE prevalence at the city level (**c**) and the county level (**d**). **e** Range of echinococcosis prevalence in cities and counties by province. Areas colored gray on the maps were not included in the survey. AE, Alveolar echinococcosis; CE, cystic echinococcosis

The national prevalence of CE and AE were 0.36% and 0.09%, respectively (Fig. 1). Echinococcosis prevalence varied substantially between provinces (see Fig. 1a, b); for example, the highest CE prevalence was detected in Tibet (1.5%), followed by Sichuan (0.90%) and Qinghai (0.67%), and the highest AE prevalence was observed in Qinghai (0.43%), followed by Sichuan (0.28%) and Tibet (0.19%). Mapping the spatial distribution of prevalence of both types of echinococcosis at the city and county levels (Fig. 2) revealed that CE was distributed across the western provinces, with high endemic areas concentrated in the Tibetan Plateau (Fig. 2a, b), while AE was mainly confined to the region of the Tibetan Plateau, with high prevalence predominant in the junction of Qinghai, Tibet, and Sichuan provinces (Fig. 2c, d). In addition, we noted that both CE and AE prevalence varied substantially at the county level within provinces (Fig. 2e) and that this variation was more apparent within provinces with a relatively high echinococcosis prevalence. For example, CE prevalence among counties in Tibet ranged from 0.11% in Luozha county, Shannan region to 7.48% in Zuogong county, Changdu region, while AE prevalence among counties in Qinghai ranged from 0% in some northern counties to 7.88% in Dari county, Guoluo region. The top five cities and top 10 counties with the highest echinococcosis prevalence are listed in Additional file 6: Tables S10, S11.

**Fig. 3:**
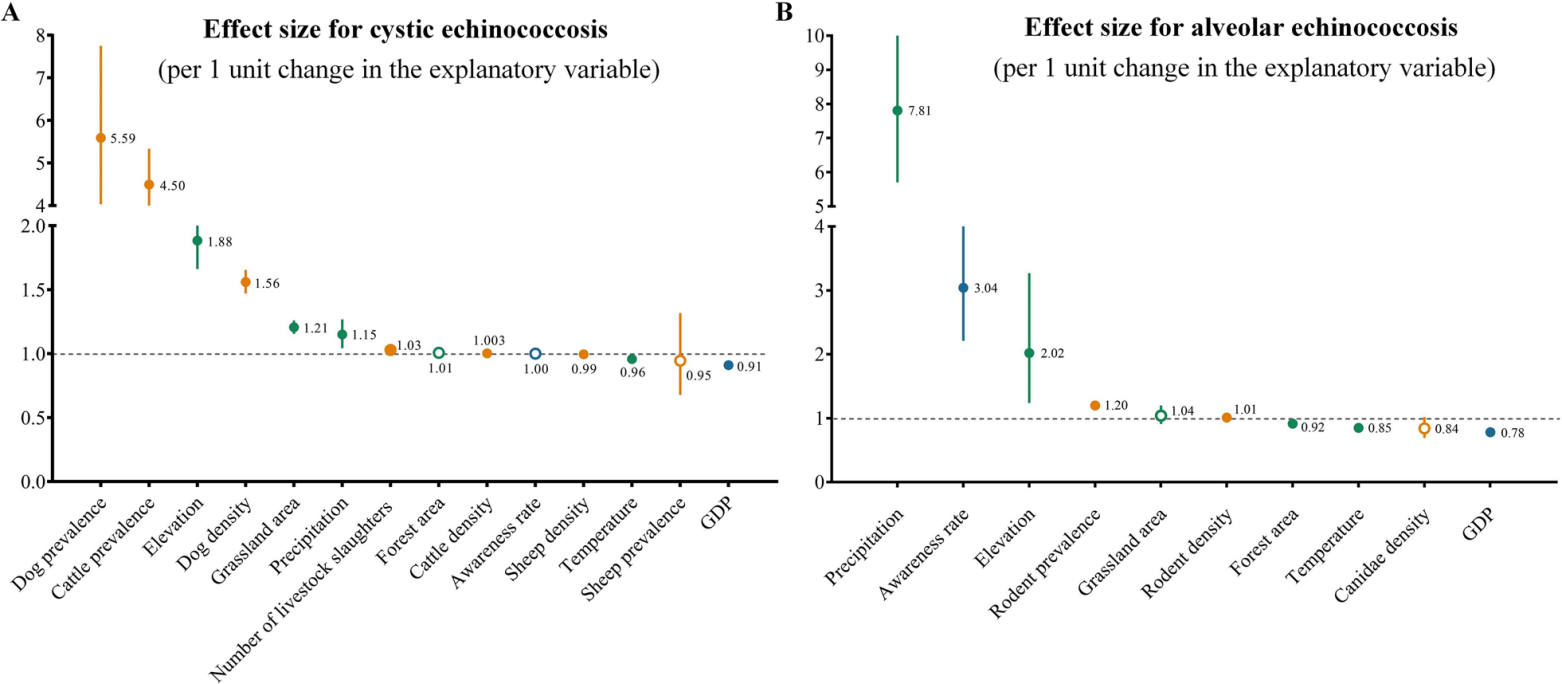
Estimates for the effect of risk factors on cystic echinococcosis (**a**) and alveolar echinococcosis (**b**). Each marker represents the estimated effect of a 1 unit increase in the explanatory variable, expressed as a change in the echinococcosis prevalence. Error bars are defined as the 95% confidence interval. Colored markers indicate the different types of factors: environmental factors (green), biological factors (yellow) and social factors (blue). Markers empty of color indicate that the variable had no significant effect on the result. GDP, Gross domestic product

To explore drivers of spatial heterogeneity in CE and AE across China, we fitted a multivariable generalized linear model to measure the association between county-level prevalence in humans and a series of environmental, biological and socio-economic factors. The statistical results and model performance are shown in Additional file 7: Table S12. We further computed effect sizes for risk factors that could influence human echinococcosis, evaluating the effect per 1 unit change in the explanatory variable and expressing the result as a change in the outcome variable (Fig. 3; Table 3).

**Table 3:** Relative risk ratios for water sources that could influence human echinococcosis

Water source		Relative risk ratio (95% CI)	
From	Compared to	Cystic echinococcosis	Alveolar echinococcosis
Tap water	Ditch	1.09 (0.91–1.31)*	1.56 (1.09–2.24)*
	River	1.57 (1.42–1.74)*	2.53 (2.08–3.08)*
	Ponding	1.40 (1.08–1.80)*	4.35 (2.23–8.48)*
	Well	1.37 (1.23–1.52)*	1.13 (0.84–1.52)
	Spring	1.57 (1.41–1.75)*	1.22 (8.59–1.72)
	Pond	1.21 (0.45–3.23)	43.8 (5.75–334)*

*CI* Confidence interval

*Significantly different from zero at *P* < 0.05 based on the 95% CI

Regarding the environmental factors, elevation and precipitation had significant positive associations with CE and AE prevalence (*P* < 0.005), while temperature was negatively associated. An 88–102% increase in echinococcosis risk occurred when elevation increased by 1 unit, whereas a 4–15% decrease in the risk of echinococcosis occurred for every 1 °C increase in temperature.

For biological factors, a 1% increase in dog and cattle prevalence was associated with around a 5.6- and 4.5-fold increase, respectively, in the prevalence of CE in humans. Dog density, cattle density and the number of livestock slaughtered had positive associations with CE prevalence (*P* < 0.0001), with a 1% increase in dog density increasing prevalence by 56%. Sheep density had a marginally negative association with CE prevalence. For AE, significantly positive associations were found with the density of rodents, as well as the prevalence of *Echinococcus* in rodents (*P* < 0.0001). We estimated that there is a 1% increase in AE risk for per 1 unit increase in rodent density, and a 20% increase in risk for every 1% change in rodent prevalence.

For social-economic factors, higher GDP was associated with lower echinococcosis risk (*P* < 0.0001), with the average effect on CE and AE being a 9% decrease and a 22% decrease, respectively, for each 1-unit increase in GDP. The types of sources of drinking water were significantly associated with both echinococcosis risks (Table 3). Compared with living close by tap water, we predicted a 10–60% increase in CE risk when living close to a ditch, river, pond, well or spring. We estimated that exposure to water from a ditch, river or pond increased the AE infection risk by 1.5- to 4.3-fold.

## Discussion

Based on the national epidemic survey for echinococcosis between 2012 and 2016, we analyzed the demographic epidemiological characteristics and geographical distribution of CE and AE in China, subsequently identifying the populations and areas at high risk of both types of echinococcosis. Moreover, we systematically and quantitatively analyzed the risk factors for CE and AE, considering a more comprehensive range of risk factors than assessed in previous studies. We not only considered demographic risk factors, but also assessed the extent to which environmental, biological and social factors would influence the echinococcosis infection risk. The results of this study may aid researchers and policy-makers in improving surveillance and preventive measures aimed at reducing *Echinococcus* infection in humans.

We illustrated the demographic characteristics of echinococcosis at national and sub-national levels. In general, our results indicated that the prevalence of echinococcosis was significantly associated with gender, age, occupation and education level. Similar to previous studies [18–20], we found that being female is a potential risk factor for CE and AE. This may be due to women being responsible for housework, including feeding dogs, collecting yak dung for fuel and milking livestock, all activities which increase their exposure frequency to *Echinococcus* spp. eggs. Moreover, we observed that CE prevalence increased with age, which may be attributable to its long incubation period and the gradual acquisition of disease in a population with age or possibly because older people have a reduced immune system. In contrast, AE prevalence declined among persons older than 60 years, which might be related to an early death among persons infected with AE. Regarding occupation, we disclosed that herdsmen and religious workers were more likely to be infected with echinococcosis than other occupations, which is in line with the findings of previous studies [21, 22]. Religious workers are at risk of echinococcosis infection because of their protection and feeding of stray dogs, while herdsmen run the same risk due to their frequent contact with both livestock and dogs. In addition, our results implied that limited education will increase the risk of echinococcosis infection, which was also reported by a number of previous studies [12, 20]. One possible explanation for this is that education level can determine occupation choice and lifestyle to a certain extent. Another possible explanation is that limited education may be linked to poor hygiene habits, such as drinking water without boiling, eating raw vegetables and not washing hands before meals. Thus, personalized and precise strategies for the prevention and control of echinococcosis should be applied to specific populations, with an emphasis on females, elderly people, herdsmen, religious workers and illiterate people.

We also mapped the spatial distribution of CE and AE at the city and county levels, and showed that the prevalence of echinococcosis differs geographically. We observed that the Tibetan Plateau region is highly co-endemic for both types of echinococcosis, which may be attributable to the combination of local environmental, biological and social conditions favoring the transmission of echinococcosis, such as, for example, high elevation, low temperature, various animals, limited health care and education and poverty. Furthermore, by combining the county-level echinococcosis cases with a range of environmental, biological and social factors, we identified and quantified the spatial risk factors for echinococcosis that might explain local geographical variations of disease using a generalized linear model.

We found that precipitation, temperature, grass or forest areas and elevation have a significant influence on both CE and AE. In line with previous studies [23, 24], our analysis indicated that both CE and AE prevalence are positively correlated with precipitation, but negatively correlated with temperature, possibly because cold, humid environmental conditions are favorable for the survival, development and reproduction of *Echinococcus* spp. eggs [25, 26]. Moreover, temperature and precipitation may further synergistically influence echinococcosis through their interplay. For example, a study carried out in Ningxia found that the risk of AE increased by 0.60% per 1-mm increase in summer mean precipitation, while it decreased by 10.60% per 1-mm increase in winter mean precipitation [27]. In addition, numerous studies have suggested a link between landscape and echinococcosis risk [28]. Giraudoux et al. found that echinococcosis prevalence is positively correlated with grassland area ratio but negatively correlated with forest area ratio [28], which is in line with our findings. This may be due to deforestation for farming created extensive shrub and grassland, which benefits the population growth in intermediate hosts (i.e. livestock and small mammals) [29, 30]. We also observed a positive correlation between elevation and echinococcosis prevalence, which is consistent with previous findings [23, 31, 32].

Our results suggested that factors related to biology play important roles in influencing echinococcosis prevalence. Specifically, we found that cattle density, cattle prevalence, dog density, dog prevalence and the number of livestock slaughtered have positive associations with CE prevalence; these results are in agreement with those of previous studies [12, 18]. Schantz et al. indicated that those persons owning livestock have a threefold higher risk of being diagnosed with this disease in comparison with those without livestock [12]. Meanwhile, a meta-analysis conducted by Alessia et al. showed that the slaughter of animals at home and dog ownership are significant risk factors associated with CE [18]. Unexpectedly, sheep density was observed to be negatively correlated with CE in our study, possibly attributable to the move of slaughtering operations from the home to slaughterhouses in areas of high sheep density, which decreases the risk of echinococcosis infection to some extent. For AE, our analysis showed that high rodent density and prevalence contributed to high disease prevalence, which is consistent with the findings of previous studies [33]. In addition, although some researchers have revealed that contact with stray dogs and wild animals was a risk factor for AE [19], unexpectedly, our results showed no significant relationship between Canidae density and AE; this may be the result of using low-resolution data of Canidae density converted from shapefiles.

In terms of social factors, we identified local economic conditions (GDP) and drinking water sources as risk factors for both CE and AE, which have also been reported as risk factors in previous research [17–19]. For example, two cross-sectional studies showed that low income increased the risk of both echinococcosis infections [18, 19]. Some studies suggest that consuming stream or river water significantly increases the echinococcosis infection risk whereas drinking tap water or well water decreases the risk [12, 17]. On the one hand, in underdeveloped economies there may be a lack of education and poor hygiene habits, resulting in an increased vulnerable to echinococcosis. Drinking water sources, on the other hand, are considered to be associated with human exposure to *Echinococcus* spp. eggs. Notably, rivers, springs, ditches and ponds are more susceptible to egg contamination than tap water because of their role in feeding various animal hosts. Surprisingly, a positive relationship was found between AE and awareness rate. This may be explained by the fact that most counties in our study had been included in the national echinococcosis control program that was launched in 2005.

Overall, our results further illustrate that control measures such as livestock management, deworming dogs, sanitation improvement and health education are essential for controlling echinococcosis; such measures have been proven to be feasible and effective in some provinces. For example, from 2017 to 2019, various measures were implemented in Tibet to strengthen the management of infection sources of echinococcosis, including dog registration and management, reduction of the dog population, monthly dog deworming and monitoring of dog infections [34]. As a result of these measures, the dog infection rate rapidly declined from 7.3% in 2016 to 1.7% in 2019, greatly reducing the risk of human infection with echinococcosis [34].

There are some limitations that should be noted in this study. First, our analysis is based on retrospective data from the national echinococcosis survey, which was carried out using portable B-ultrasonography to examine the subjects. Only the abdominal lesions of echinococcosis could be detected, whereas lesions in the lungs, brain or other areas could not be detected. Thus, the reported prevalence in this survey among people may be underestimated. Second, several studies have proved that wild animals, such as foxes and wolves, can be infected with *E. multilocularis* [35]; however, fine-scale data were not available on the wild animals in China due to practical difficulties in the field survey. Third, some researchers take the complex interaction among environmental, biological and social factors into consideration [31], which has not been discussed in this study. Therefore, a further study with more focus on complex interactions among various risk factors is suggested.

## Conclusion

Based on the most extensive and authoritative echinococcosis data derived from the national epidemiological survey between 2012 and 2016, our study provides a comprehensive understanding of demographic characteristics, geographical patterns and risk factors of both CE and AE in China. We computed sex-, age group-, occupation- and education level-specific prevalences of both diseases at national and sub-national levels, and found that female gender, older age, occupation as herdsman, occupation as religious worker and illiteracy were risk factors for both types of echinococcosis. We mapped the spatial patterns of CE and AE at city and county levels, showing that CE was distributed across western provinces, with high endemic areas concentrated in the Tibetan Plateau while AE was mainly confined to the region of the Tibetan Plateau, with high prevalence predominant in the junction of Qinghai, Tibet and Sichuan provinces. In addition, we fitted generalized linear models to measure the association between county-level prevalence in humans and a series of environmental, biological and socio-economic factors. Our results indicated that CE was positively correlated with cattle density, cattle prevalence, dog density, dog prevalence, number of livestock slaughtered, elevation and grass area, and negatively associated with temperature and GDP. AE was positively correlated with precipitation, level of awareness, elevation, rodent density and rodent prevalence, and negatively correlated with forest area, temperature and GDP. In summary, our study results have the potential to benefit targeted prevention and control of the disease. Furthermore, the epidemiological analysis methods used in this study can provide a reference for analysis of echinococcosis data in other countries or other diseases with similar prevalence and risk data.

## Supplementary Information

**Additional file 1.** **Questionnaire 1**. Household basic information questionnaire. **Questionnaire 2**. Knowledge for echinococcosis prevention and control.

**Additional file 2.** **Figure S1**. The spatial distribution of environmental, biological and social factors adopted in this study. **Figure S2**. Correlation and variance inflation factorof variables adopted in cystic echinococcosis modeling. **Figure S3**. Correlation and variance inflation factorof variables adopted in alveolar echinococcosis modeling. **Figure S4**. Sex-age specific distribution of cystic echinococcosisand alveolar echinococcosisat nation level. **Figure S5**.  Sex-age specific distribution of cystic echinococcosisand alveolar echinococcosisby province.

**Additional file 3.** **Text S1**. Odds ratio computation.

**Additional file 4.** **Text S2**. Model analysis.

**Additional file 5.** **Table S1**. Demographic characteristics of human Echinococcosis in Inner Mongolia. **Table S2**. Demographic characteristics of human Echinococcosis in Sichuan. **Table S3**. Demographic characteristics of human Echinococcosis in Yunnan. **Table S4**. Demographic characteristics of human Echinococcosis in Tibet. **Table S5**. Demographic characteristics of human Echinococcosis in Shaanxi. **Table S6**. Demographic characteristics of human Echinococcosis in Gansu. **Table S7**. Demographic characteristics of human Echinococcosis in Qinghai. **Table S8**. Demographic characteristics of human Echinococcosis in Ningxia. **Table S9**. Demographic characteristics of human Echinococcosis in Xinjiang.

**Additional file 6.** **Table S10**. Top 5 cities of cystic and alveolar echinococcosis prevalence in China. **Table S11**. Top 10 counties of cystic and alveolar echinococcosis prevalence in China.

**Additional file 7.** **Table S12**. Statistic results of GLM model for cystic and alveolar echinococcosis.

## Data Availability

All data generated or analysed during this study are included in this published article (and its electronic supplementary material files).

## Abbreviations

AE: Alveolar echinococcosis
CE: Cystic echinococcosis
CGIAR: Consultative Group on International Agricultural Research
China CDC: Chinese Center for Disease Control and Prevention
CI: Confidence interval
CMDS: China Meteorological Data Service Center
DALYs: Disability-adjusted life-years
ELISA: Enzyme-linked immunosorbent assay
GDP: Gross domestic product
OR: Odds ratio
RESDC: The Data Center for Resources and Environmental Sciences, Chinese Academy of Science
SEDAC: The Socioeconomic Data and Applications Center
VIF: Variance inflation factor
