# Supplementary material for: Epidemiological characteristics and risk factors for cystic and alveolar echinococcosis in China: an analysis of a national population-based field survey
Source: Parasit Vectors. 2023 Jun 3;16:181. doi: 10.1186/s13071-023-05788-z (PMC10239570; doi:10.1186/s13071-023-05788-z)
Supplement: Supplementary file 1 — Additional file 1. Questionnaire 1. Household basic information questionnaire. Questionnaire 2. Knowledge for echinococcosis prevention and control. [file 13071_2023_5788_MOESM1_ESM.docx]

**Questionnaire 1:**

Household basic information questionnaire

Province: _ City:_ County:_ Address:_

| Household ID | Number of dogs raised | Number of sheep raised | Number of cattle raised | Number of livestock slaughtered | drinking water source |
| --- | --- | --- | --- | --- | --- |
|  |  |  |  |  |  |
|  |  |  |  |  |  |
| …… | …… | …… | …… | …… | …… |
|  |  |  |  |  |  |

Note:

Drink water source: 1. Ditch; 2. River; 3. Well; 4. Pond; 5. Ponding; 6. Spring; 7. Tap water.

**Questionnaire 2:**

**Knowledge for echinococcosis prevention and control**

ID: _ Sex: _ Age: _ Address: _ Date: _

Question 1: How did people get infected with echinococcosis?

1. Eat raw beef and mutton b) dogs infection c) have no idea.

Question 2: Do you wash your hands before eating?

1. Yes b) sometimes c) no

Question 3: Do you play with dogs?

1. Frequently b) sometimes c) never

Question 4: How to deal with the sick liver or lung of livestock after slaughter?

1. Throw away b) eat c) feed dogs d) Burial or incineration

Question 5: Would you like to deworm dogs for free?

1. yes b) no

Note: For each question, you can choose more than one answer. 20 points for each question, and 60 points marks qualified.
