# Supplementary material for: Epidemiological characteristics and risk factors for cystic and alveolar echinococcosis in China: an analysis of a national population-based field survey
Source: Parasit Vectors. 2023 Jun 3;16:181. doi: 10.1186/s13071-023-05788-z (PMC10239570; doi:10.1186/s13071-023-05788-z)

**Figure S1.** The spatial distribution of environmental, biological and social factors adopted in this study.


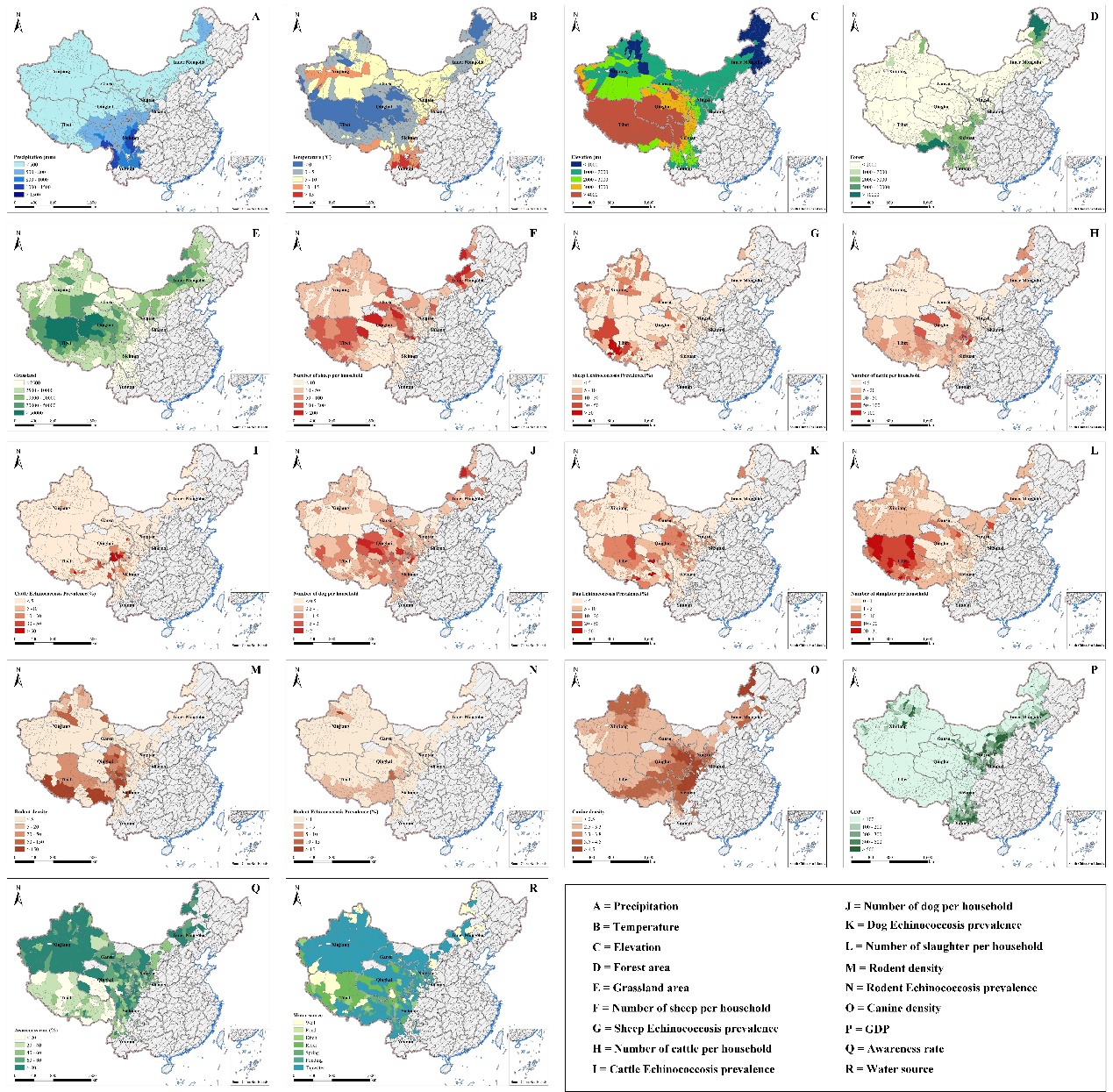


**Figure S2.** Correlation and variance inflation factor (VIF) of variables adopted in cystic echinococcosis modelling.


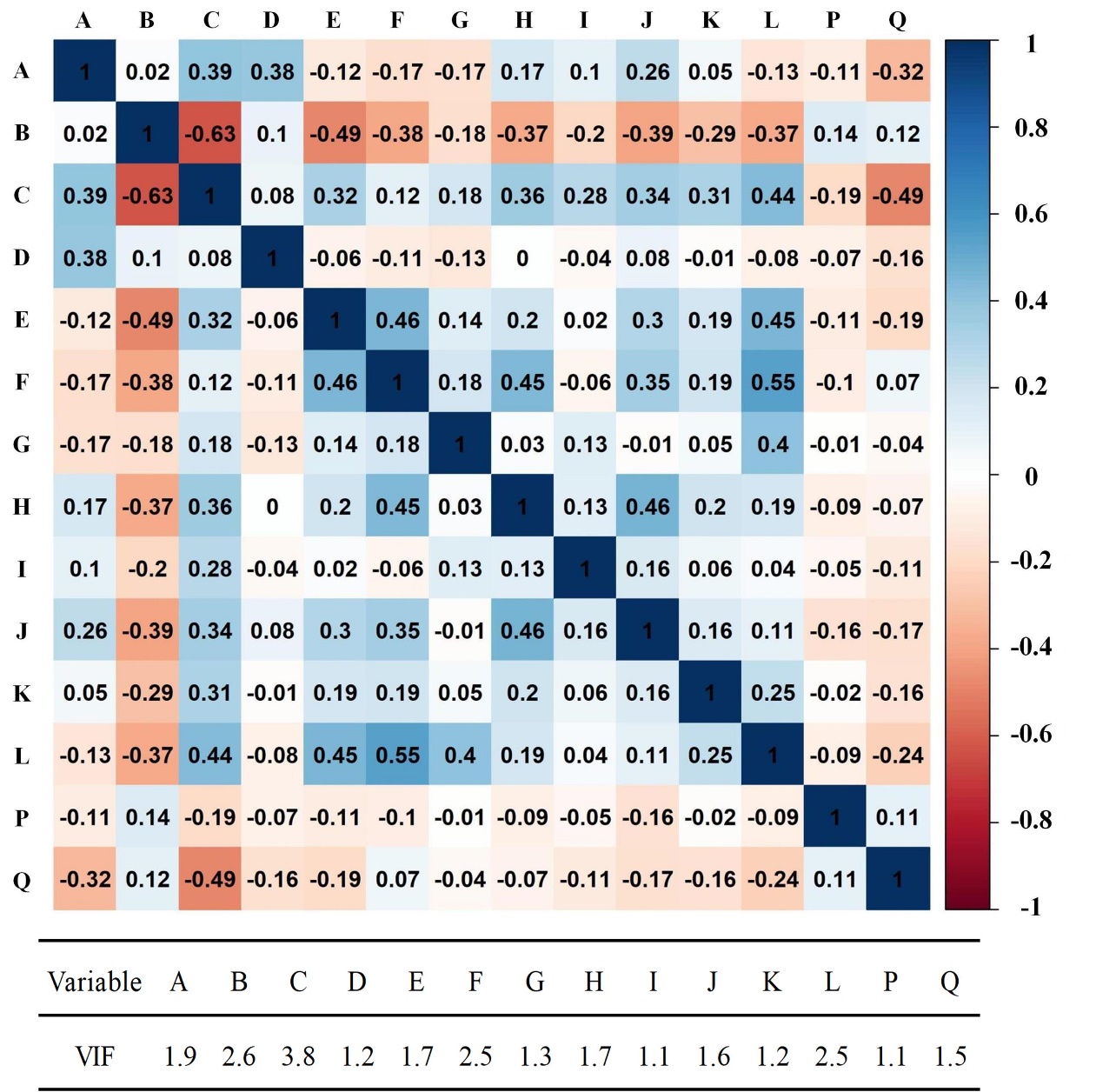


**Figure S3.** Correlation and variance inflation factor (VIF) of variables adopted in alveolar echinococcosis modelling.


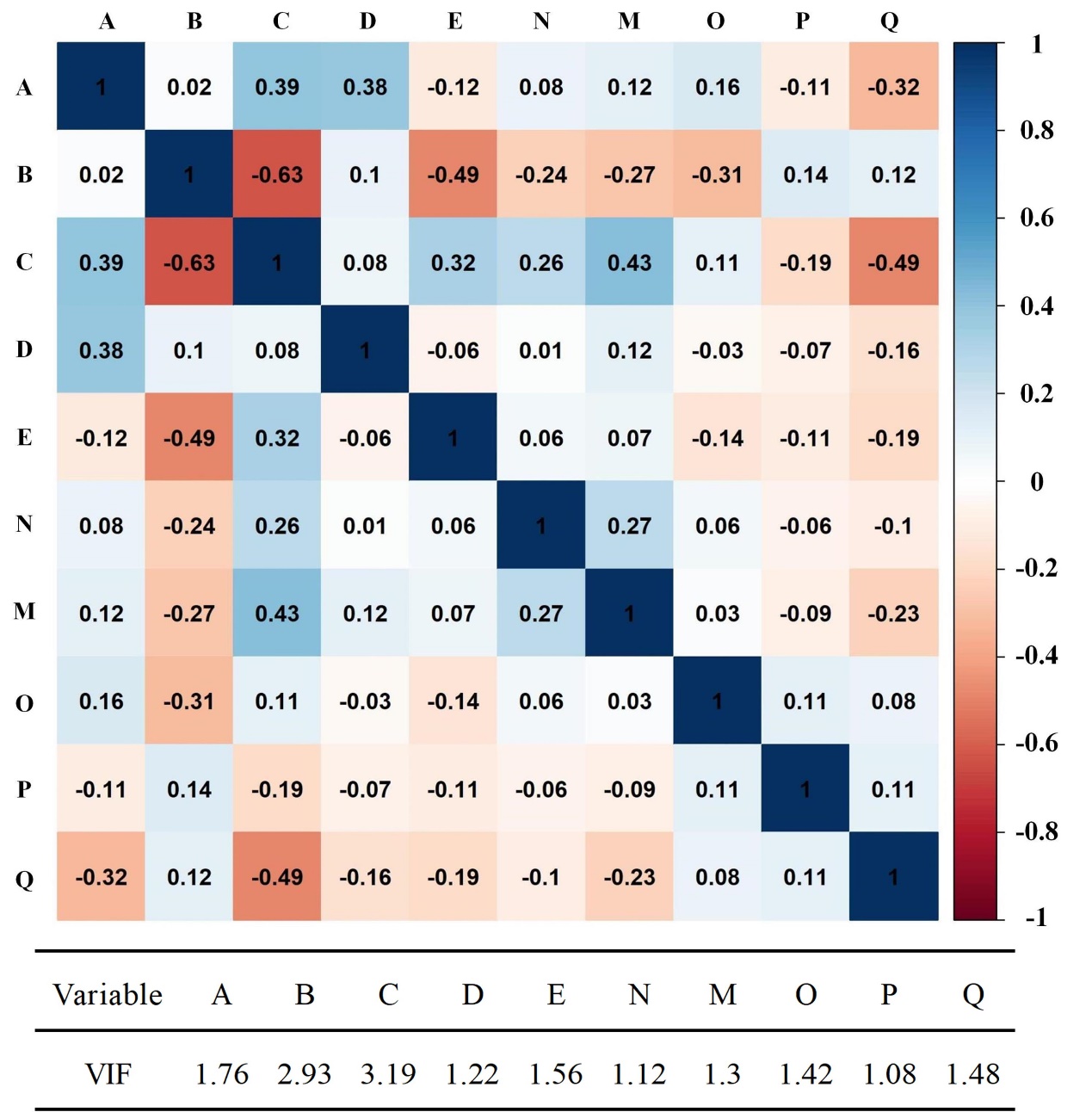


**Figure S4.** Sex-age specific distribution of cystic echinococcosis (A) and alveolar echinococcosis (B) at nation level.


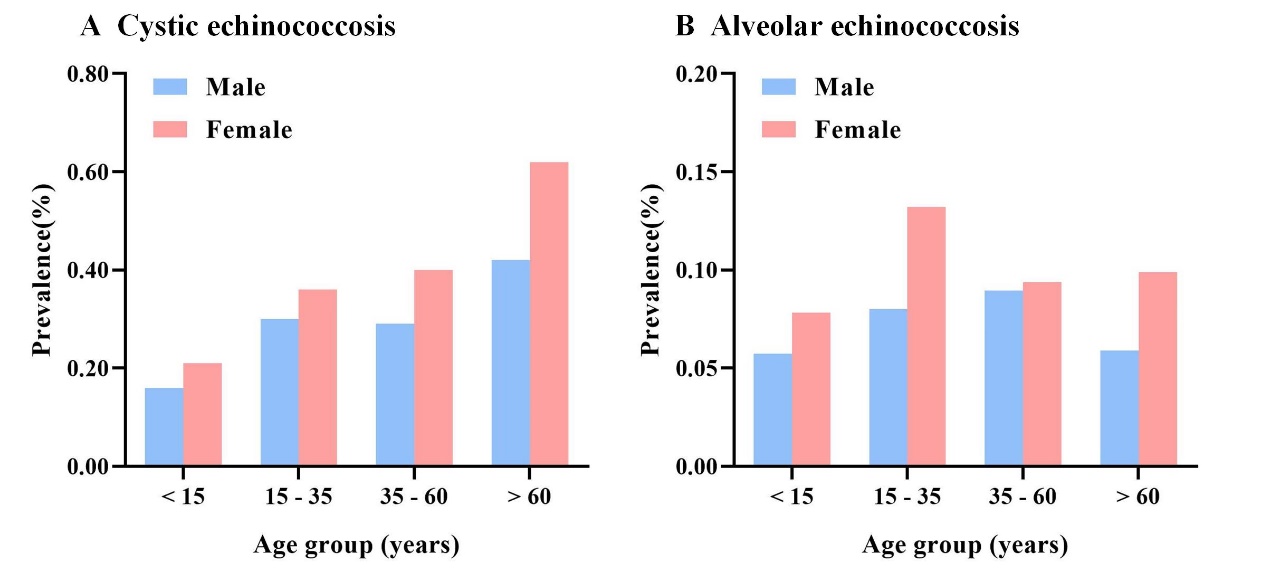


**Figure S5.** Sex-age specific distribution of cystic echinococcosis (A) and alveolar echinococcosis (B) by province.


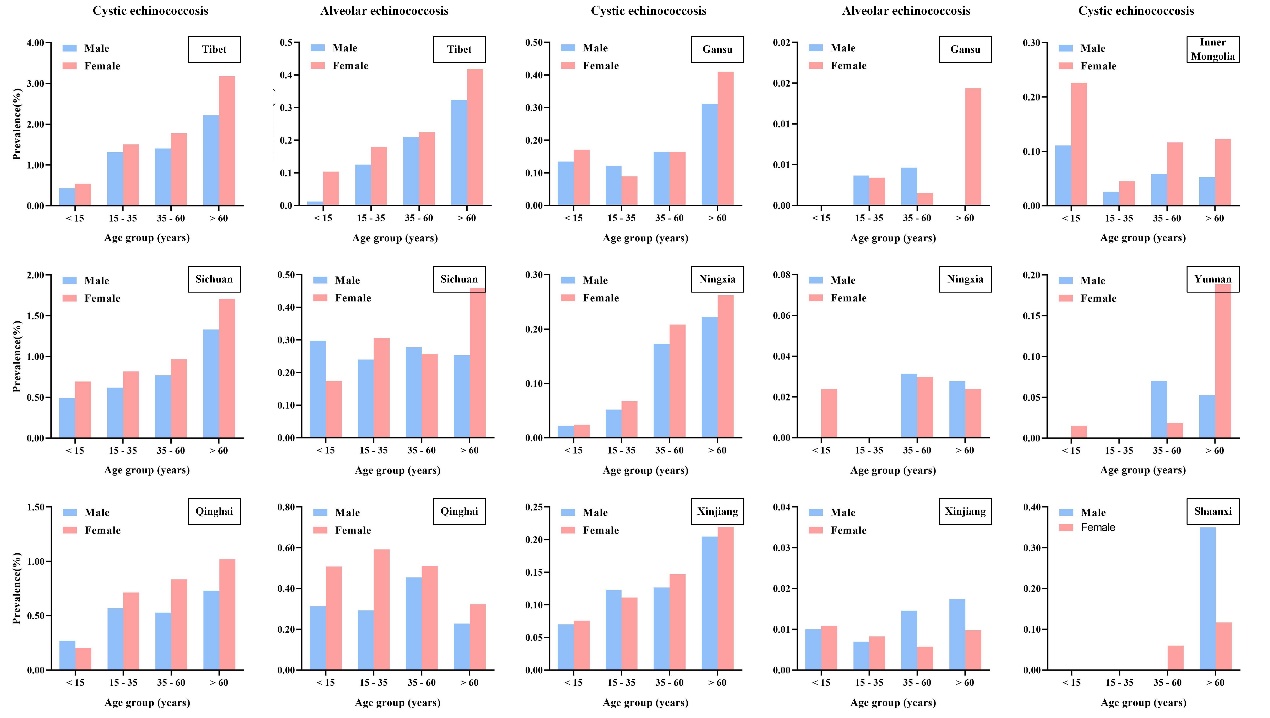

Supplement: Supplementary file 2 — Additional file 2. Figure S1. The spatial distribution of environmental, biological and social factors adopted in this study. Figure S2. Correlation and variance inflation factorof variables adopted in cystic echinococcosis modeling. Figure S3. Correlation and variance inflation factorof variables adopted in alveolar echinococcosis modeling. Figure S4. Sex-age specific distribution of cystic echinococcosisand alveolar echinococcosisat nation level. Figure S5. Sex-age specific distribution of cystic echinococcosisand alveolar echinococcosisby province. [file 13071_2023_5788_MOESM2_ESM.docx]
