# Supplementary material for: Epidemiological characteristics and risk factors for cystic and alveolar echinococcosis in China: an analysis of a national population-based field survey
Source: Parasit Vectors. 2023 Jun 3;16:181. doi: 10.1186/s13071-023-05788-z (PMC10239570; doi:10.1186/s13071-023-05788-z)
Supplement: Supplementary file 3 — Additional file 3. Text S1. Odds ratio computation. [file 13071_2023_5788_MOESM3_ESM.docx]

**Text S1.** Odds ratio (OR) computation.

| **OR Framework** | | **Disease Infected** | |
| --- | --- | --- | --- |
|  |  | Yes | No |
| **Exposed** | Yes | a | b |
|  | No | c | d |

$$OR=\frac{a}{c}/\frac{b}{d}=ad/bc$$
