# Supplementary material for: Epidemiological characteristics and risk factors for cystic and alveolar echinococcosis in China: an analysis of a national population-based field survey
Source: Parasit Vectors. 2023 Jun 3;16:181. doi: 10.1186/s13071-023-05788-z (PMC10239570; doi:10.1186/s13071-023-05788-z)
Supplement: Supplementary file 4 — Additional file 4. Text S2. Model analysis. [file 13071_2023_5788_MOESM4_ESM.docx]

**Text S2.** Model analysis.

We first transformed all data into the same geographic coordinate system (WGS-84) and the same projected coordinate system (Albers conical area), then we matched the county-level prevalence of echinococcosis with a range of environmental, biological, and social factors by county code. Finally, we fitted a generalized linear model with poisson distribution to measure the association between county-level echinococcosis prevalence and a series of covariates (Eq. 1).

$Y_{i}=\alpha+Log(N_{i})+X_{E_{i}}+X_{B_{i}}+X_{S_{i}}+\varepsilon$ Eq 1

The dependent variable $Y_{i}$ was the number of cystic echinococcosis (or alveolar echinococcosis) cases in county I, which was assumed to follow a poission distribution; $\alpha$denotes the intercept; $\varepsilon$was defined as the error term; $Log(N_{i})$is the logarithm of the population examined for county i, which is included as an offset to normalize the echinococcosis cases data.$X_{E_{i}}$was the environmental factors in county i, including precipitation, temperature, grass area, forest area, and elevation; $X_{B_{i}}$ indicated biological factors in county i. Considering the differences in transmission cycle and animal host between cystic echinococcosis and alveolar echinococcosis, different animal hosts were selected as biological factors suitable for each disease model. For cystic echinococcosis, we chose the density and prevalence of cattle and sheep, dog density and prevalence, and slaughter numbers as biological factors. While rodent density, rodent prevalence, and Canidae density were selected as biological factors for alveolar echinococcosis. $X_{S_{i}}$represents social factors in county i, including GDP, awareness rate, and drinking water source. In addition, the covariates of precipitation, elevation, forest area, grassland area, and GDP were log transformed.
