# Supplementary material for: Epidemiological characteristics and risk factors for cystic and alveolar echinococcosis in China: an analysis of a national population-based field survey
Source: Parasit Vectors. 2023 Jun 3;16:181. doi: 10.1186/s13071-023-05788-z (PMC10239570; doi:10.1186/s13071-023-05788-z)
Supplement: Supplementary file 5 — Additional file 5. Table S1. Demographic characteristics of human Echinococcosis in Inner Mongolia. Table S2. Demographic characteristics of human Echinococcosis in Sichuan. Table S3. Demographic characteristics of human Echinococcosis in Yunnan. Table S4. Demographic characteristics of human Echinococcosis in Tibet. Table S5. Demographic characteristics of human Echinococcosis in Shaanxi. Table S6. Demographic characteristics of human Echinococcosis in Gansu. Table S7. Demographic characteristics of human Echinococcosis in Qinghai. Table S8. Demographic characteristics of human Echinococcosis in Ningxia. Table S9. Demographic characteristics of human Echinococcosis in Xinjiang. [file 13071_2023_5788_MOESM5_ESM.docx]

**Table S1**. Demographic characteristics of human Echinococcosis in Inner Mongolia.

| Characteristic | No. of total examined | CE | | |
| --- | --- | --- | --- | --- |
|  |  | No. positive | Prevalence (%) | Odds ratio (95% CI) |
| **Sex^*^** | | | | |
| Male | 42,313 | 22 | 0.05 | 1 |
| Female | 47,782 | 51 | 0.11 | 2.05 (1.25 – 3.39) |
| **Age (years)^*^** | | | | |
| < 15 | 1,786 | 3 | 0.17 | 1 |
| 15~35 | 16,688 | 6 | 0.04 | 0.21 (0.05 – 0.86) |
| 35~60 | 47,936 | 43 | 0.09 | 0.53 (0.16 – 1.72) |
| > 60 | 23,685 | 21 | 0.09 | 0.53 (0.16 – 1.77) |
| **Occupation^*^** | | | | |
| Herdsmen | 28,671 | 36 | 0.13 | 1.97 (1.10 – 3.56) |
| Semi-farmers-and-herders | 2,533 | 1 | 0.04 | 0.62 (0.08 – 4.68) |
| Farmers | 33,728 | 19 | 0.06 | 0.88 (0.46 – 1.72) |
| Religious personage | 6 | 0 | 0.00 | NA |
| Others | 25,156 | 16 | 0.06 | 1 |
| **Degree of education^*^** | | | | |
| Preschool | 281 | 1 | 0.36 | 3.23 (0.42 – 24.90) |
| Illiterate | 10,854 | 12 | 0.11 | 1 |
| Primary school | 29,406 | 37 | 0.13 | 1.14 (0.59 – 2.18) |
| Junior high school | 35,407 | 18 | 0.05 | 0.46 (0.22 – 0.95) |
| Senior high school | 9,166 | 3 | 0.03 | 0.29 (0.08 – 1.05) |
| College or above | 4,981 | 2 | 0.04 | 0.36 (0.08 – 1.62) |

**Table S2.** Demographic characteristics of human Echinococcosis in Sichuan.

| Characteristic | No. of total examined | CE | | | AE | | |
| --- | --- | --- | --- | --- | --- | --- | --- |
|  |  | No. positive | Prevalence (%) | Odds ratio  (95% CI) | No. positive | Prevalence (%) | Odds ratio  (95% CI) |
| **Sex^*^** | | | | | | | |
| Male | 53,088 | 411 | 0.77 | 1 | 140 | 0.26 | 1 |
| Female | 59,517 | 602 | 1.01 | 1.31 (1.15 - 1.48) | 177 | 0.30 | 1.13 (0.90 – 1.41) |
| **Age (years)^*^** | | | | | | | |
| < 15 | 9,320 | 55 | 0.59 | 1 | 22 | 0.24 | 1 |
| 15~35 | 36,452 | 263 | 0.72 | 1.22 (0.91 – 1.64) | 100 | 0.27 | 1.16 (0.73 – 1.85) |
| 35~60 | 50,191 | 439 | 0.87 | 1.49 (1.12 – 1.97) | 134 | 0.27 | 1.13 (0.72 – 1.78) |
| > 60 | 16,642 | 256 | 1.54 | 2.63 (1.96 – 3.53) | 61 | 0.37 | 1.55 (0.95 – 2.53) |
| **Occupation^*^** | | | | | | | |
| Herdsmen | 24,933 | 649 | 2.60 | 5.41 (4.07 – 7.21) | 263 | 1.05 | 7.37 (4.38 – 12.41) |
| Semi-farmers-and-herders | 9,532 | 79 | 0.83 | 1.69 (1.19 – 2.41) | 8 | 0.08 | 0.85 (0.36 – 2.00) |
| Farmers | 67,488 | 227 | 0.34 | 0.68 (0.50 – 0.93) | 24 | 0.04 | 0.25 (0.13 – 0.47) |
| Religious personage | 268 | 7 | 2.61 | 5.43 (2.44 – 12.09) | 7 | 2.61 | 18.54 (7.50 – 45.85) |
| Others | 10,384 | 51 | 0.49 | 1 | 15 | 0.14 | 1 |
| **Degree of education^*^** | | | | | | | |
| Preschool | 1,189 | 5 | 0.42 | 0.27 (0.11 – 0.66) | 2 | 0.17 | 0.35 (0.08 – 1.40) |
| Illiterate | 48,422 | 732 | 1.51 | 1 | 233 | 0.48 | 1 |
| Primary school | 42,899 | 236 | 0.55 | 0.36 (0.31 – 0.41) | 75 | 0.17 | 0.36 (0.28 – 0.47) |
| Junior high school | 15,724 | 29 | 0.18 | 0.12 (0.08 – 0.17) | 6 | 0.04 | 0.08 (0.03 – 0.18) |
| Senior high school | 3,724 | 8 | 0.22 | 0.14 (0.07 – 0.28) | 0 | 0.000 | NA |
| College or above | 647 | 3 | 0.46 | 0.30 (0.10 – 0.94) | 1 | 0.16 | 0.32 (0.04 – 2.29) |

**Table S3.** Demographic characteristics of human Echinococcosis in Yunnan.

| Characteristic | No. of total examined | CE | | |
| --- | --- | --- | --- | --- |
|  |  | No. positive | Prevalence (%) | Odds ratio(95% CI) |
| **Sex** | | | | |
| Male | 57,885 | 21 | 0.04 | 1 |
| Female | 66,228 | 31 | 0.05 | 1.29 (0.74 – 2.25) |
| **Age (years)^*^** | | | | |
| < 15 | 27,564 | 2 | 0.01 | 1 |
| 15~35 | 24,425 | 0 | 0.00 | NA |
| 35~60 | 49,943 | 21 | 0.04 | 5.80 (1.36 – 24.73) |
| > 60 | 22,220 | 29 | 0.13 | 18.01 (4.30 – 75.48) |
| **Occupation** | | | | |
| Herdsmen | 0 | 0 | 0.00 | NA |
| Semi-farmers-and-herders | 78 | 0 | 0.00 | NA |
| Farmers | 95,586 | 50 | 0.05 | 0.88 (0.46 – 1.72) |
| Religious personage | 1 | 0 | 0.00 | NA |
| Others | 28,481 | 2 | 0.01 | 1 |
| **Degree of education^*^** | | | | |
| Preschool | 3,372 | 0 | 0.00 | NA |
| Illiterate | 22,157 | 20 | 0.09 | 1 |
| Primary school | 64,539 | 22 | 0.03 | 0.38 (0.21 – 0.69) |
| Junior high school | 28,987 | 10 | 0.03 | 0.38 (0.18 – 0.82) |
| Senior high school | 3,809 | 0 | 0.00 | NA |
| College or above | 1,192 | 0 | 0.00 | NA |

**Table S4.** Demographic characteristics of human Echinococcosis in Tibet.

| Characteristic | No. of total examined | CE | | | AE | | |
| --- | --- | --- | --- | --- | --- | --- | --- |
|  |  | No. positive | Prevalence (%) | Odds ratio  (95% CI) | No. positive | Prevalence (%) | Odds ratio  (95% CI) |
| **Sex^*^** | | | | | | | |
| Male | 34,297 | 427 | 1.25 | 1 | 53 | 0.15 | 1 |
| Female | 46,087 | 775 | 1.68 | 1.36 (1.20 - 1.53) | 100 | 0.22 | 1.40 (1.01 – 1.96) |
| **Age (years)^*^** | | | | | | | |
| < 15 | 15,870 | 77 | 0.49 | 1 | 9 | 0.06 | 1 |
| 15~35 | 21,053 | 300 | 1.43 | 2.96 (2.31 – 3.81) | 33 | 0.16 | 2.77 (1.32 – 5.78) |
| 35~60 | 33,468 | 546 | 1.63 | 3.40 (2.68 – 4.32) | 73 | 0.22 | 3.85 (1.93 – 7.70) |
| > 60 | 9,993 | 279 | 2.79 | 5.89 (4.57 – 7.59) | 38 | 0.38 | 6.73 (3.25 – 13.92) |
| **Occupation^*^** | | | | | | | |
| Herdsmen | 18,558 | 604 | 0.00 | 5.40 (4.45 – 6.56) | 63 | 0.00 | 4.26 (2.46 – 7.38) |
| Semi-farmers-and-herders | 10,078 | 145 | 1.44 | 2.34 (1.84 – 2.98) | 29 | 0.29 | 3.61 (1.96 – 6.65) |
| Farmers | 31,369 | 317 | 1.01 | 1.64 (1.33 – 2.02) | 45 | 0.14 | 1.80 (1.02 – 3.18) |
| Religious personage | 345 | 12 | 3.48 | 5.79 (3.17 – 10.57) | 0 | 0.00 | NA |
| Others | 20,034 | 124 | 0.62 | 1 | 16 | 0.08 | 1 |
| **Degree of education^*^** | | | | | | | |
| Preschool | 6,496 | 12 | 0.18 | 0.08 (0.05 – 0.15) | 1 | 0.02 | 0.06 (0.01 – 0.43) |
| Illiterate | 39,616 | 844 | 2.13 | 1 | 101 | 0.25 | 1 |
| Primary school | 26,331 | 261 | 0.99 | 0.46 (0.40 – 0.53) | 37 | 0.14 | 0.55 (0.38 – 0.80) |
| Junior high school | 5,383 | 58 | 1.08 | 0.50 (0.40 – 0.53) | 10 | 0.19 | 0.72 (0.38 – 1.39) |
| Senior high school | 970 | 14 | 1.44 | 0.67 (0.40 – 1.15) | 2 | 0.21 | 0.81 (0.20 – 3.28) |
| College or above | 1,588 | 13 | 0.82 | 0.38 (0.22 – 0.66) | 2 | 0.13 | 0.49 (0.12 – 2.00) |

**Table S5.** Demographic characteristics of human Echinococcosis in Shaanxi.

| Characteristic | No. of total examined | CE | | |
| --- | --- | --- | --- | --- |
|  |  | No. positive | Prevalence (%) | Odds ratio (95% CI) |
| **Sex** | | | | |
| Male | 2,896 | 3 | 0.10 | 1 |
| Female | 3,284 | 2 | 0.06 | 0.59 (0.10 – 3.52) |
| **Age (years)^*^** | | | | |
| < 15 | 257 | 0 | 0.00 | 1 |
| 15~35 | 1,186 | 0 | 0.00 | NA |
| 35~60 | 3,021 | 1 | 0.03 | NA |
| > 60 | 1,711 | 4 | 0.23 | NA |
| **Occupation** | | | | |
| Herdsmen | 0 | 0 | 0.00 | NA |
| Semi-farmers-and-herders | 0 | 0 | 0.00 | NA |
| Farmers | 5,685 | 5 | 0.09 | NA |
| Religious personage | 0 | 0 | 0.00 | NA |
| Others | 495 | 0 | 0.00 | 1 |
| **Degree of education^*^** | | | | |
| Preschool | 80 | 0 | 0.00 | NA |
| Illiterate | 2,469 | 3 | 0.12 | 1 |
| Primary school | 1,683 | 1 | 0.06 | 0.49 (0.05 – 4.70) |
| Junior high school | 1,470 | 1 | 0.07 | 0.56 (0.06 – 5.38) |
| Senior high school | 340 | 0 | 0.00 | NA |
| College or above | 131 | 0 | 0.00 | NA |

**Table S6.** Demographic characteristics of human Echinococcosis in Gansu.

| Characteristic | No. of total examined | CE | | | AE | | |
| --- | --- | --- | --- | --- | --- | --- | --- |
|  |  | No. positive | Prevalence (%) | Odds ratio  (95% CI) | No. positive | Prevalence (%) | Odds ratio  (95% CI) |
| **Sex^*^** | | | | | | | |
| Male | 123,486 | 228 | 0.18 | 1 | 4 | 0.003 | 1 |
| Female | 127,048 | 255 | 0.20 | 1.09 (0.91 - 1.30) | 6 | 0.005 | 1.46 (0.41 – 5.17) |
| **Age (years)^*^** | | | | | | | |
| < 15 | 9,896 | 15 | 0.15 | 1 | 0 | 0.000 | 1 |
| 15~35 | 56,605 | 59 | 0.10 | 0.69 (0.39 – 1.21) | 2 | 0.004 | NA |
| 35~60 | 129,863 | 213 | 0.16 | 1.08 (0.64 – 1.83) | 4 | 0.003 | NA |
| > 60 | 54,170 | 196 | 0.36 | 2.39 (1.41 – 4.04) | 4 | 0.007 | NA |
| **Occupation^*^** | | | | | | | |
| Herdsmen | 18,856 | 135 | 0.72 | 6.85 (4.74 – 9.89) | 0 | 0.000 | NA |
| Semi-farmers-and-herders | 9,559 | 23 | 0.24 | 2.29 (1.36 – 3.87) | 0 | 0.000 | NA |
| Farmers | 187,886 | 289 | 0.15 | 1.46 (1.03 – 2.07) | 9 | 0.005 | 1.64 (0.21 – 12.94) |
| Religious personage | 13 | 0 | 0.00 | NA | 0 | 0.000 | NA |
| Others | 34,220 | 36 | 0.11 | 1 | 1 | 0.003 | 1 |
| **Degree of education^*^** | | | | | | | |
| Preschool | 885 | 0 | 0.00 | NA | 0 | 0.000 | NA |
| Illiterate | 57,075 | 189 | 0.33 | 1 | 7 | 0.012 | 1 |
| Primary school | 96,413 | 195 | 0.20 | 0.61 (0.50 – 0.75) | 1 | 0.001 | 0.08 (0.01 – 0.68) |
| Junior high school | 74,594 | 78 | 0.10 | 0.32 (0.24 – 0.41) | 2 | 0.003 | 0.22 (0.05 – 1.05) |
| Senior high school | 15,155 | 16 | 0.11 | 0.32 (0.19 – 0.53) | 0 | 0.000 | NA |
| College or above | 6,412 | 5 | 0.08 | 0.23 (0.10 – 0.57) | 0 | 0.000 | NA |

**Table S7.** Demographic characteristics of human Echinococcosis in Qinghai.

| Characteristic | No. of total examined | CE | | | AE | | |
| --- | --- | --- | --- | --- | --- | --- | --- |
|  |  | No. positive | Prevalence (%) | Odds ratio  (95% CI) | No. positive | Prevalence (%) | Odds ratio  (95% CI) |
| **Sex^*^** | | | | | | | |
| Male | 58,348 | 322 | 0.55 | 1 | 208 | 0.36 | 1 |
| Female | 63,335 | 495 | 0.78 | 1.42 (1.23 - 1.63) | 323 | 0.51 | 1.43 (1.20 – 1.71) |
| **Age (years)^*^** | | | | | | | |
| < 15 | 8,410 | 20 | 0.24 | 1 | 34 | 0.40 | 1 |
| 15~35 | 41,119 | 266 | 0.65 | 2.73 (1.73 - 4.30) | 186 | 0.45 | 1.12 (0.78 - 1.61) |
| 35~60 | 53,766 | 369 | 0.69 | 2.90 (1.85 - 4.55) | 260 | 0.48 | 1.20 (0.84 - 1.71) |
| > 60 | 18,388 | 162 | 0.88 | 3.73 (2.34 - 5.94) | 51 | 0.28 | 0.69 (0.44 - 1.06) |
| **Occupation^*^** | | | | | | | |
| Herdsmen | 44,982 | 665 | 0.00 | 4.91 (3.88 - 6.20) | 437 | 0.97 | 4.98 (3.73 - 6.65) |
| Semi-farmers-and-herders | 3,862 | 13 | 0.00 | 1.10 (0.61 - 1.99) | 13 | 0.34 | 1.71 (0.93 - 3.15) |
| Farmers | 45,890 | 35 | 0.08 | 0.25 (0.17 - 0.37) | 3 | 0.01 | 0.03 (0.01 - 0.11) |
| Religious personage | 1,024 | 25 | 2.44 | 8.19 (5.20 - 12.89) | 27 | 2.64 | 13.74 (8.58 - 22.00) |
| Others | 25,925 | 79 | 0.30 | 1 | 51 | 0.20 | 1 |
| **Degree of education^*^** | | | | | | | |
| Preschool | 1,818 | 1 | 0.06 | 0.04 (0.01 - 0.32) | 1 | 0.06 | 0.07 (0.01 - 0.48) |
| Illiterate | 36,283 | 450 | 1.24 | 1 | 291 | 0.80 | 1 |
| Primary school | 53,226 | 303 | 0.57 | 0.46 (0.39 - 0.53) | 204 | 0.38 | 0.47 (0.40 - 0.57) |
| Junior high school | 19,577 | 31 | 0.16 | 0.13 (0.09 - 0.18) | 17 | 0.09 | 0.11 (0.06 - 0.17) |
| Senior high school | 6,355 | 10 | 0.16 | 0.12 (0.07 - 0.23) | 2 | 0.03 | 0.04 (0.01 - 0.16) |
| College or above | 4,422 | 22 | 0.50 | 0.40 (0.26 - 0.61) | 16 | 0.36 | 0.45 (0.27 - 0.74) |

**Table S8**. Demographic characteristics of human Echinococcosis in Ningxia.

| Characteristic | No. of total examined | CE | | | AE | | |
| --- | --- | --- | --- | --- | --- | --- | --- |
|  |  | No. positive | Prevalence (%) | Odds ratio  (95% CI) | No. positive | Prevalence (%) | Odds ratio  (95% CI) |
| **Sex^*^** | | | | | | | |
| Male | 30,267 | 42 | 0.14 | 1 | 6 | 0.02 | 1 |
| Female | 41,685 | 71 | 0.17 | 1.23 (0.84 - 1.80) | 9 | 0.02 | 1.09 (0.39 – 3.06) |
| **Age (years)^*^** | | | | | | | |
| < 15 | 8,730 | 2 | 0.02 | 1 | 1 | 0.01 | 1 |
| 15~35 | 14,729 | 9 | 0.06 | 2.67 (0.58 - 12.35) | 0 | 0.00 | NA |
| 35~60 | 32,903 | 64 | 0.19 | 8.51 (2.08 - 34.75) | 10 | 0.03 | 2.43 (0.31 - 19.00) |
| > 60 | 15,590 | 38 | 0.24 | 10.66 (2.57 - 44.21) | 4 | 0.03 | 2.24 (0.25 - 20.05) |
| **Occupation^*^** | | | | | | | |
| Herdsmen | 0 | 0 | 0.00 | NA | 0 | 0.00 | NA |
| Semi-farmers-and-herders | 0 | 0 | 0.00 | NA | 0 | 0.00 | NA |
| Farmers | 52,590 | 105 | 0.20 | 5.52 (2.57 - 11.86) | 13 | 0.02 | 2.34 (0.53 - 10.39) |
| Religious personage | 42 | 1 | 2.38 | 67.29 (8.096 - 559.26) | 0 | 0.00 | NA |
| Others | 19,320 | 7 | 0.04 | 1 | 2 | 0.01 | 1 |
| **Degree of education^*^** | | | | | | | |
| Preschool | 1,121 | 0 | 0.00 | NA | 0 | 0.00 | NA |
| Illiterate | 18,159 | 52 | 0.29 | 1 | 5 | 0.03 | 1 |
| Primary school | 28,441 | 44 | 0.15 | 0.54 (0.36 – 0.81) | 7 | 0.02 | 0.89 (0.28 – 2.82) |
| Junior high school | 18,186 | 8 | 0.04 | 0.15 (0.07 – 0.32) | 1 | 0.01 | 0.20 (0.02 – 1.71) |
| Senior high school | 4,479 | 6 | 0.13 | 0.47 (0.20 – 1.09) | 2 | 0.04 | 1.62 (0.31 – 8.36) |
| College or above | 1,566 | 3 | 0.19 | 0.67 (0.21 – 2.14) | 0 | 0.00 | NA |

**Table S9.** Demographic characteristics of human Echinococcosis in Xinjiang.

| Characteristic | No. of total examined | CE | | | AE | | |
| --- | --- | --- | --- | --- | --- | --- | --- |
|  |  | No. positive | Prevalence (%) | Odds ratio  (95% CI) | No. positive | Prevalence (%) | Odds ratio  (95% CI) |
| **Sex^*^** | | | | | | | |
| Male | 144711 | 194 | 0.13 | 1 | 18 | 0.01 | 1 |
| Female | 148422 | 209 | 0.14 | 1.05 (0.86 - 1.28) | 11 | 0.01 | 0.60 (0.28 – 1.26) |
| **Age (years)^*^** | | | | | | | |
| < 15 | 19243 | 14 | 0.07 | 1 | 2 | 0.01 | 1 |
| 15~35 | 91650 | 107 | 0.12 | 1.61 (0.92 - 2.80) | 7 | 0.01 | 0.73 (0.15 – 3.54) |
| 35~60 | 138760 | 190 | 0.14 | 1.88 (1.09 – 3.24) | 14 | 0.01 | 0.97 (0.22 – 4.27) |
| > 60 | 43480 | 92 | 0.21 | 2.91 (1.66 - 5.11) | 6 | 0.01 | 1.33 (0.27 – 6.58) |
| **Occupation^*^** | | | | | | | |
| Herdsmen | 22074 | 60 | 0.00 | 2.51 (1.80 – 3.49) | 4 | 0.00 | 4.73 (1.06 – 21.13) |
| Semi-farmers-and-herders | 6231 | 10 | 0.00 | 1.48 (0.77 – 2.85) | 1 | 0.00 | 4.19 (0.44 – 40.27) |
| Farmers | 186491 | 248 | 0.13 | 1.22 (0.96 - 1.57) | 21 | 0.01 | 2.94 (0.88 – 9.85) |
| Religious personage | 62 | 0 | 0.00 | NA | 0 | 0.00 | NA |
| Others | 78275 | 85 | 0.11 | 1 | 3 | 0.004 | 1 |
| **Degree of education^*^** | | | | | | | |
| Preschool | 5035 | 3 | 0.06 | 0.24 (0.07 – 0.76) | 0 | 0.00 | NA |
| Illiterate | 15470 | 39 | 0.25 | 1 | 2 | 0.01 | 1 |
| Primary school | 112196 | 155 | 0.14 | 0.55 (0.39 – 0.78) | 20 | 0.02 | 1.38 (0.32 – 5.90) |
| Junior high school | 115712 | 145 | 0.13 | 0.50 (0.35 – 0.71) | 5 | 0.004 | 0.33 (0.06 – 1.72) |
| Senior high school | 27884 | 34 | 0.12 | 0.48 (0.30 – 0.77) | 0 | 0.00 | NA |
| College or above | 16836 | 27 | 0.16 | 0.64 (0.39 – 1.04) | 2 | 0.01 | 0.92 (0.13- 6.52) |
