# Supplementary material for: Epidemiological characteristics and risk factors for cystic and alveolar echinococcosis in China: an analysis of a national population-based field survey
Source: Parasit Vectors. 2023 Jun 3;16:181. doi: 10.1186/s13071-023-05788-z (PMC10239570; doi:10.1186/s13071-023-05788-z)
Supplement: Supplementary file 6 — Additional file 6. Table S10. Top 5 cities of cystic and alveolar echinococcosis prevalence in China. Table S11. Top 10 counties of cystic and alveolar echinococcosis prevalence in China. [file 13071_2023_5788_MOESM6_ESM.docx]

**Table S10**. Top 5 cities of cystic and alveolar echinococcosis prevalence in China.

| CE |  |  |  | AE |  |  |  |
| --- | --- | --- | --- | --- | --- | --- | --- |
| Province | City/Region | No.of positive/total | Prevalence (%) | Province | City/Region | No.of positive/total | Prevalence (%) |
| Tibet | Shannan | 59/897 | 6.58 | Qinghai | Guoluo | 427/15890 | 2.59 |
| Qinghai | Yushu | 221/7025 | 3.15 | Qinghai | Yushu | 100/7025 | 1.42 |
| Tibet | Naqu | 339/11897 | 2.85 | Sichuan | Ganzi | 296/58121 | 0.51 |
| Qinghai | Guoluo | 412/15890 | 2.59 | Tibet | Shannan | 4/897 | 0.45 |
| Tibet | Ali | 94/4740 | 1.98 | Tibet | Naqu | 339/11897 | 0.28 |

**Table S11.** Top 10 counties of cystic and alveolar echinococcosis prevalence in China.

| CE | | | | | AE | | | | |
| --- | --- | --- | --- | --- | --- | --- | --- | --- | --- |
| Province | City/Region | County | No.of positive/total | Prevalence (%) | Province | City/Region | County | No.of positive/total | Prevalence (%) |
| Tibet | Changdu | Zuogong | 66/882 | 7.48 | Qinghai | Guoluo | Dari | 284/3605 | 7.88 |
| Sichuan | Ganzi | Shiqu | 229/3198 | 7.16 | Sichuan | Ganzi | Shiqu | 143/318 | 4.47 |
| Tibet | Shannan | Cuomei | 59/897 | 6.58 | Qinghai | Guoluo | Banma | 91/2466 | 3.69 |
| Tibet | Naqu | Anduo | 44/833 | 5.28 | Qinghai | Yushu | Chengduo | 87/2444 | 3.56 |
| Qinghai | Guoluo | Gande | 171/3256 | 5.25 | Sichuan | Ganzi | Seda | 70/3199 | 2.19 |
| Qinghai | Yushu | Zhiduo | 28/561 | 4.99 | Qinghai | Guoluo | Maduo | 2/135 | 1.48 |
| Sichuan | Ganzi | Seda | 151/3199 | 4.72 | Sichuan | Ganzi | Ganzi | 34/3200 | 1.06 |
| Qinghai | Yushu | Zaduo | 42/933 | 4.50 | Tibet | Naqu | Baqing | 8/823 | 0.97 |
| Tibet | Naqu | Baqing | 37/823 | 4.49 | Tibet | Ali | Cuoqin | 8/824 | 0.97 |
| Qinghai | Guoluo | Dari | 154/3605 | 4.27 | Tibet | Naqu | Suo | 7/812 | 0.86 |
