# Supplementary material for: Epidemiological characteristics and risk factors for cystic and alveolar echinococcosis in China: an analysis of a national population-based field survey
Source: Parasit Vectors. 2023 Jun 3;16:181. doi: 10.1186/s13071-023-05788-z (PMC10239570; doi:10.1186/s13071-023-05788-z)
Supplement: Supplementary file 7 — Additional file 7. Table S12. Statistic results of GLM model for cystic and alveolar echinococcosis. [file 13071_2023_5788_MOESM7_ESM.docx]

**Table S12**. Statistic results of GLM model for cystic and alveolar echinococcosis.

|  | **Cystic echinococcosis** | | | | **Alveolar echinococcosis** | | | |
| --- | --- | --- | --- | --- | --- | --- | --- | --- |
|  | Estimate | Std | Z value | P value | Estimate | Std | Z value | P value |
| Intercept | -13.180 | 0.562 | -23.433 | 0.000^***^ | -25.650 | 2.058 | -12.462 | 0.000^***^ |
| Precipitation (log) | 0.139 | 0.050 | 2.806 | 0.005^**^ | 2.055 | 0.161 | 12.809 | 0.000^***^ |
| Temperature | -0.043 | 0.008 | -5.778 | 0.000^***^ | -0.164 | 0.024 | -6.791 | 0.000^***^ |
| Elevation (log) | 0.634 | 0.064 | 9.899 | 0.000^***^ | 0.701 | 0.247 | 2.834 | 0.005^**^ |
| Forest (log) | 0.007 | 0.007 | 0.910 | 0.363 | -0.089 | 0.019 | -4.617 | 0.000^***^ |
| Grassland (log) | 0.187 | 0.022 | 8.565 | 0.000^***^ | 0.044 | 0.070 | 0.624 | 0.532 |
| Sheep density | -0.004 | 0.0004 | -11.869 | 0.000^***^ |  |  |  |  |
| Cattle density | 0.003 | 0.0006 | 4.727 | 0.000^***^ |  |  |  |  |
| Dog density | 0.445 | 0.030 | 14.765 | 0.000^***^ |  |  |  |  |
| Sheep prevalence | -0.056 | 0.169 | -0.331 | 0.740 |  |  |  |  |
| Cattle prevalence | 1.503 | 0.087 | 17.174 | 0.000^***^ |  |  |  |  |
| Dog prevalence | 1.721 | 0.167 | 10.331 | 0.000^***^ |  |  |  |  |
| Number of slaughter | 0.029 | 0.005 | 5.562 | 0.000^***^ |  |  |  |  |
| Canidae density |  |  |  |  | -0.173 | 0.099 | -1.751 | 0.080 |
| Rodent density |  |  |  |  | 0.008 | 0.000 | 18.182 | 0.000^***^ |
| Rodent prevalence |  |  |  |  | 0.180 | 0.014 | 13.323 | 0.000^***^ |
| GDP (log) | -0.094 | 0.015 | -6.218 | 0.000^***^ | -0.247 | 0.042 | -5.886 | 0.000^***^ |
| Awareness rate (%) | 0.001 | 0.001 | 1.328 | 0.184 | 1.112 | 0.163 | 6.843 | 0.000^***^ |
| Tap water (ref) | -0.008 | 0.454 | -0.017 | 0.986 | -9.952 | 328.2 | -0.030 | 0.976 |
| Ditch (factor) | 0.087 | 0.092 | 0.945 | 0.344 | 0.444 | 0.184 | 2.408 | 0.016 |
| River (factor) | 0.452 | 0.050 | 8.903 | 0.000^***^ | 0.928 | 0.100 | 9.277 | 0.000^***^ |
| Ponding (factor) | 0.334 | 0.130 | 2.573 | 0.01^*^ | 1.469 | 0.341 | 4.308 | 0.000^***^ |
| Well (factor) | 0.313 | 0.054 | 5.828 | 0.000^***^ | 0.119 | 0.152 | 0.781 | 0.435 |
| Spring (factor) | 0.453 | 0.056 | 8.113 | 0.000^***^ | 0.196 | 0.177 | 1.104 | 0.269 |
| Pond (factor) | 0.187 | 0.504 | 0.372 | 0.710 | 3.780 | 1.036 | 3.647 | 0.000^***^ |
| AIC | 4978.1 | | | | 1539.4 |  |  |  |
| R2 | 0.641 | |  |  | 0.900 |  |  |  |
| Adj-R2 | 0.621 | |  |  | 0.890 |  |  |  |
